# Sharing Responsibilities within the General Practice Team – A Cross-Sectional Study of Task Delegation in Germany

**DOI:** 10.1371/journal.pone.0157248

**Published:** 2016-06-09

**Authors:** Karola Mergenthal, Martin Beyer, Ferdinand M. Gerlach, Corina Guethlin

**Affiliations:** Institute of General Practice, Goethe-University Frankfurt, Frankfurt/Main, Germany; National Institute of Health, ITALY

## Abstract

**Background:**

Expected growth in the demand for health services has generated interest in the more effective deployment of health care assistants. Programs encouraging German general practitioners (GPs) to share responsibility for care with specially qualified health care assistants in the family practice (VERAHs) have existed for several years. But no studies have been conducted on the tasks German GPs are willing to rely on specially qualified personnel to perform, what they are prepared to delegate to all non-physician practice staff and what they prefer to do themselves.

**Methods:**

As part of an evaluation study on the deployment of VERAHs in GP-centered health care, we used a questionnaire to ask about task delegation within the practice team. From a list of tasks that VERAHs are specifically trained to carry out, GPs were asked to indicate which they actually delegate. We also asked GPs why they had employed a VERAH in their practice and for their opinions on the benefits and limitations of assigning tasks to VERAHs. The aim of the study was to find out which tasks GPs delegate to their specially qualified personnel, which they permit all HCAs to carry out, and which tasks they do not delegate at all.

**Results:**

The survey was filled in and returned by 245 GPs (83%). Some tasks were exclusively delegated to VERAHs (e.g. home visits), while others were delegated to all HCAs (e.g. vaccinations). About half the GPs rated the assessment of mental health, as part of the comprehensive assessment of a patient’s condition, as the sole responsibility of a GP.

The possibility to delegate more complex tasks was the main reason given for employing a VERAH. Doctors said the delegation of home visits provided them with the greatest relief.

**Conclusions:**

In Germany, where GPs are solely accountable for the health care provided in their practices, experience with the transfer of responsibility to other non-physician health care personnel is still very limited. When HCAs have undergone special training, GPs seem to be prepared to delegate tasks that demand a substantial degree of know-how, such as home visits and case management. This “new” role allocation within the practice may signal a shift in the provision of health care by family practice teams in Germany.

## Introduction

In an aging population, it is increasingly difficult for primary care providers to deliver appropriate health care to a rising number of chronically ill patients. In some countries, it is standard practice to employ qualified nurses in primary care. In Germany, however, GPs generally employ health care assistants (HCAs) who work under the supervision of a GP. Health care assistants are increasingly being trained to take on additional responsibilities, but GPs remain legally accountable for the health care provided in their practices. HCAs that receive additional training are referred to as “health care assistants in the family practice (VERAHs)” [1]. In other countries they are called “medical assistants” [2], “physician assistants” [3], “healthcare assistants” [4], or in a broader sense “allied health assistants” [5]. The overarching aim is to increase the efficiency of comprehensive health care by delegating tasks, and thus sharing responsibility. Medical assistants are traditionally given rather minor tasks to do, such as measuring blood glucose, and applying wound dressings [2]. VERAHs, however, are trained to take on a new role: Following their initial qualification (lasting 3 years), they undergo an extra 200 hours of training during which they explicitly learn to perform more of the tasks needed in the care of chronically ill patients, such as carrying out routine home visits, team-based case management, supporting patients in the coordination and organization of their treatment, assessment of physical and mental health as part of a comprehensive assessment, certain tasks in preventive medicine, and wound care.

The professional training of an HCA in Germany lasts three years, of which 1–2 days a week are spent at a vocational school an d3-4 days in a practice. HCAs perform clinical tasks such as taking blood samples, intramuscular injections, ECGs and spirometries. After working for 2 years, it is possible to obtain the VERAH qualification (it costs 1,850–2,600 € to qualify as a VERAH). GPs that have signed a contract with one of the major health insurance plans in Baden-Wuerttemberg are compensated for employing a VERAH. VERAHs are expected to perform the tasks named above, but the GP still decides which tasks to delegate and to whom.

From a legal perspective, GPs in Germany remain accountable for all health care services provided in their practices, regardless of whether they share the responsibility (the word responsibility being used in a broad sense throughout this document) for performing certain tasks with other practice staff. In such a health care system, delegation means assigning specific tasks to qualified health care personnel but remaining responsible and legally accountable for the health care of a patient.

In Germany, although VERAHs are required to explain and monitor medication as part of routine care management, many tasks, such as advising patients how and when to take their medicine, are still regarded as the responsibility of the physician. The introduction of new roles demands an understanding of alternative ways of organizing team-based care, and the outcomes that may result. Data for Germany is scarce, but available information shows that jointly performed interventions (by GP and HCA) can improve the care of patients with osteoarthritis, depression and heart failure [6–8]. Team-based care can result in higher patient satisfaction [9], reduction of acute care utilization [10], greater compliance and a closer bond to the general practice team [11].

Furthermore, practice teams see the new responsibilities of HCAs as an effective resource that leads to a general improvement in health care, particularly of chronically ill patients [12–13]. Studies from abroad have also shown that doctors hope that hiring physician assistants will give them more time for complex patients and reduce stress levels at work [14].

Uncertainty with regard to funding and which tasks to delegate, as well as the assistants’ need for supervision and training, has resulted in widespread skepticism [14]. A systematic review summarizing the new responsibilities and advantages and disadvantages of employing allied health assistants shows that the main benefit appears to be an improvement in service quality as a result of an increase in patient orientation, while disadvantages concern mainly role confusion due to unclear responsibilities [5]. Role confusion may also reflect physician inexperience in sharing responsibility for certain tasks with new healthcare personnel.

In order to study the transfer of responsibilities within the practice team (consisting of the GP, a specially trained HCA, or VERAH, and other HCAs) we explicitly asked GPs to describe the tasks they routinely delegate. The aim of the study was to explore which tasks GPs delegate to their specially qualified personnel, which they permit all HCAs to carry out, and which tasks they do not delegate at all (i.e. tasks they carry out themselves, or are not carried out in the practice at all). We also looked at the factors encouraging GPs to employ a VERAH in their practices and asked about the benefits and limitations of assigning tasks to VERAHs.

## Materials and Methods

### Ethics approval

The study was approved in June 2011 by the ethical review committee of the University Hospital, Goethe-University Frankfurt, Frankfurt am Main, Germany (Geschafts-Nr.:263/11).

### Study population

The study population consisted solely of general practitioners, who had participated in a special “structured health plan” (GP-centered care, Hausarztzentrierte Versorgung, HzV) in the federal state of Baden-Wuerttemberg. Baden-Wuerttemberg covers a surface area of 35 million square kilometers and has 10 million inhabitants. It has 10 towns with a population of over 100,000.

The “structured health plan” aims to enhance health care for patients with chronic diseases and complex health care needs (e.g., those requiring long-term care). Participation in the “structural health plan” is voluntary, but it includes additional reimbursement for the care of chronically ill patients, provided that the practice employs a VERAH.

The recruitment took place in two steps. Firstly, we invited all of the 909 practices that had participated in the structured health plan in 2011, and employed a VERAH, to take part in the survey. The invitation included study information, a declaration of consent, a questionnaire and a stamped addressed envelope to return the study documents. Secondly, we invited the VERAHs to give the GPs in the practice an information package containing a special questionnaire (see S1 File) for them to fill out. The study commenced between July and September 2011.

### Survey instrument

The survey was conducted on the basis of a self-developed questionnaire that focused on the reasons for and the consequences of employing a VERAH, as well as on delegation in general. From a list of the tasks (e.g. Home visits, wound management, vaccination management) that the VERAHs are specifically trained to carry out, the GPs were asked to indicate which they actually delegate to a VERAH, which to all non-physician staff, and which they generally perform themselves, The GPs were additionally asked to provide information in free text on the main advantages and disadvantages of employing a VERAH.

One item in the survey asked about the reasons for employing a VERAH (1. The possibility to improve the health care of chronically ill patients; 2. The possibility to save time; 3. The possibility to delegate home visits). Another item asked about general changes resulting from this type of team-based care.

The items included in the questionnaire were decided upon following the analysis of data derived from focus groups we conducted with HCAs, and from 2 interviews carried out with GPs. It was pilot tested in 10 general practices.

### Statistical analysis

The software program IBM SPSS (Statistics Version 20.0) was used to analyze the data descriptively. Missing data could be deducted from giving N in all tables. N includes only complete data sets. Information provided in free text was evaluated using semi-qualitative content analysis, i.e. categories were created, and statements were coded and counted.

## Results

### Characteristics of participating general practitioners

VERAHs at 294 practices participated in the survey. Physicians from 245 of these practices also took part in the survey, corresponding to a response rate of 83.3%. The average age of participating physicians was 54 years (SD 8.5), more than three quarters of them were male (77%), and they had worked in general practice for an average of 18 years (SD 8.5). Almost three quarters of the participating practices were rural (73%) which differed from all GPs in the GP-centered health care program (see Table 1). Overall, an average of 1.82 GPs (SD 1.25) and 4.68 HCAs (SD 2.25) worked in each practice, and around half of the practices were solo-practices (56%) (see Table 1). The HCAs with the additional qualification (VERAHs) had worked in their practices for an average of 12.5 years (SD 8.6).

**Table 1:** Characteristics of participating general practitioners in the state of Baden-Wuerttemberg.

Family physicians (n = 245)	
Male gender (n; %)	184 (77.3)
Age in years (Mean; SD)	54.0 (7.48)
Years in private practice (Mean; SD)	18 (8.5)
Previous participation in research project (yes) (n; %)	108 (48.4)
Family practices (n = 237)	
Solo practice (n,%)	138 (56.3)
Joint practice (n,%)	99 (43.7)
Location of practice	
City (>100,000 inhabitants) (n; %)	18 (7.7)
Town (20,000–100,000 inhabitants) (n; %)	45 (19,2)
Rural town (5,000–20,000 inhabitants) (n; %)	106 (45.3)
Village (<5,000 Einwohner (n; %)	65 (27.8)
Practice personnel	
Number of doctors in practice (Mean; SD)	1.82 (1.25)
Number of non-physician staff (Mean; SD)	4.68 (2.25)

### Delegation

#### Tasks assigned to VERAHs and other practice staff

63% of participating physicians said they relied on VERAHs to conduct structured assessments of a patient’s physical condition, 59% of the GPs said VERAHs were exclusively responsible for carrying out home visits. 58% of GPs said that only VERAHs prepared patients’ care plans, and 49% said VERAHs were solely responsible for conducting case management assessments.

Geriatric assessments were delegated either to VERAHs (44%), or to all staff members (38%).

Documenting medically significant facts is either delegated to VERAHs or carried out by the GPs themselves: 34% delegate this task to a VERAH and 34% do it themselves.

Other tasks (e.g. measurement of diagnostic parameters, simple medical procedures or vaccinations) were delegated to all practice staff (see Table 2).

**Table 2:** Tasks not delegated at all, delegated mainly to VERAHs, and delegated to all non-physician staff.

Tasks	Tasks delegated mainly to VERAHs (%,n)	Tasks delegated to all staff (%,n)	Tasks not delegated at all (%,n)
Structured assessments of a patient’s physical condition (n = 233)	62.7% (146)	15.5% (36)	21.9% (51)
Home visits (n = 235)	58.7% (138)	30.6% (72)	10.6% (25)
Geriatric assessment (n = 209)	43.5% (91)	38.3% (80)	18.2% (38)
Medically significant facts (n = 226)	34.1% (77)	29.4% (72)	34.1% (77)
Structured assessments of a patient’s mental health (n = 235)	30.2% (71)	23.4% (55)	46.4% (109)
Wound management (n = 238)	38.2% (91)	52.5% (142)	9.2% (22)
Patient training (n = 166)	36.7% (61)	51.2% (85)	12.0% (20)
Symptoms of complaint and/or disease (n = 214)	34.6% (74)	37.4% (80)	28.0% (60)
Medication management (n = 235)	29.8% (70)	51.1% (120)	19.1% (45)
Vaccination management (n = 238)	26.1% (62)	59.7% (142)	14.3% (34)
Simple medical procedures (n = 238)	18.5% (44)	79.8% (190)	1.7% (4)
Measurement of diagnostic parameters (n = 239)	17.2% (41)	80.8% (193)	2.1% (5)
**Case Management**			
Preparation of care plan (n = 196)	58.2% (114)	14.3% (28)	27.6% (54)
Re-assessment (n = 204)	45.1% (92)	33.8% (69)	21.1% (43)
Assessment (n = 218)	49.1% (107)	31.2% (68)	19.7% (43)
Support patient in coordination and organization of treatment (n = 236)	32.6% (77)	52.1% (123)	15.3% (36)
Dialog with other institutions (n = 235)	36.6% (86)	51.9% (122)	11.5% (27)

Of the medical tasks for which the VERAHs had been specifically trained, only the assessment of mental health as part of a comprehensive assessment of a patient’s condition continued to be performed by almost half (46%) of the participating physicians (see Table 2).

Almost half the participating physicians (46%) continued to carry out the assessment of mental health as part of a comprehensive assessment of their patients’ condition themselves (see Table 2).

### Reasons for employing a VERAH

Three-quarters (74%) of the surveyed doctors said they saw the employment of a VERAH as an opportunity to improve health care for their chronically ill patients. 72% regarded it as a chance to save time, and 64% as an opportunity to delegate home visits.

**Fig 1:**
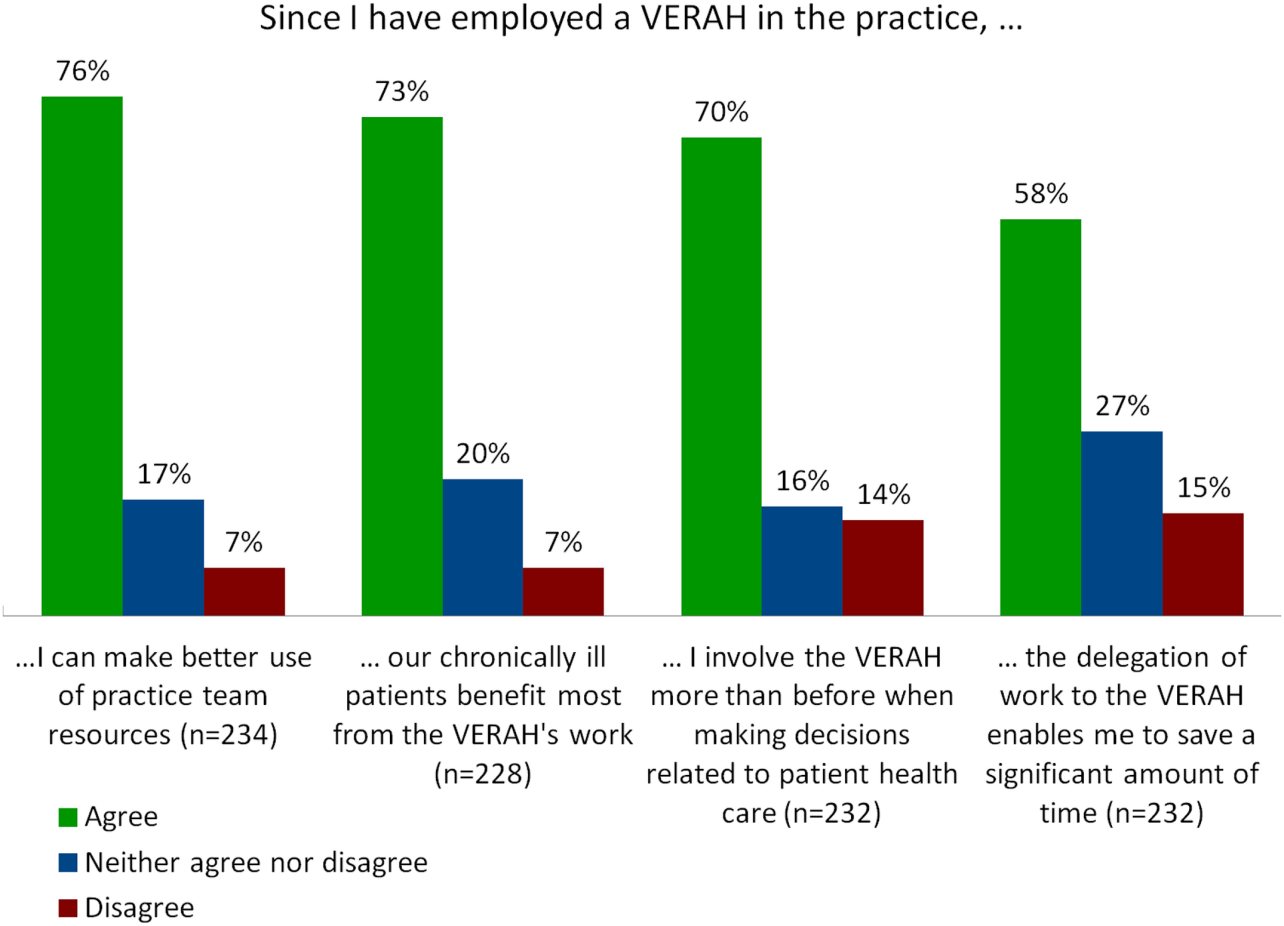
Changes resulting from the employment of a VERAH.

When asked about changes resulting from the involvement of a VERAH in team-based health care, more than 70% of GPs agreed that the involvement of a VERAH provided the opportunity to assign practice resources more efficiently and reckoned that chronically ill patients in particular benefited from this type of care. About 60% of the GPs felt that it provided them with the chance to save a significant amount of time. When asked about changes to the decision-making process, 70% agreed that they involved VERAHs in decisions affecting patient care more than they had previously (see Fig 1).

The analysis of free text fields asking about the advantages and disadvantages of employing a VERAH revealed that the delegation of home visits to VERAHs provided the greatest relief to the doctor in his everyday work (n = 58 of 240 entries, 24%). The doctor’s workload also fell as a result of an increase in the responsibilities borne by VERAHs (n = 38), as, for example, they were able to work with greater independence and assess the patient’s condition better. The delegation of simple medical tasks, the optimization of practice work processes, an improvement in the health care of the chronically ill and time savings were mentioned almost as often (from n = 32 to n = 35 times). Only n = 11 doctors said the employment of VERAHs resulted in no relief in their everyday workload. The reasons given for this were that the VERAHs were not yet taking on the additional responsibilities foreseen for them, or that the VERAHs had already been well trained before receiving the additional qualifications and had taken on greater responsibilities in patient care previous to the additional training (see examples of free text entries in Table 3).

**Table 3:** Advantages and disadvantages of employing a VERAH.

Category	Entries (n = 240)	Examples of advantages
Delegation of home visits	n = 58 (24%)	Home visits for the assessment of a patient’s condition and follow-up, for the care of elderly and/or chronically ill persons, for diagnostic tests, for routine check-ups; fewer home visits performed by doctor and no deterioration in quality
Increase in VERAH’s responsibility	n = 38 (16%)	Enhance motivation of VERAHs, thus increasing quality; greater responsibility relieves burden on doctor; greater independence for VERAH → better assessment of patient’s condition; greater professionalism →improved safety; the VERAH as a competent partner
Improve work processes in practice	n = 35 (15%)	Better organization of work processes in practice; earlier communication of changes; improvements in all areas → better documentation and organization; VERAH improves process organization to take account of the course of complex diseases; better networking
Delegation of simple medical tasks	n = 34 (14%)	Time savings resulting from delegation of simple medical tasks, e.g., taking blood samples, checking medications, wound treatment, vaccinations; delegation of needs-based assessment
Improvement in care for chronically ill	n = 32 (13%)	Improvement in care makes it easier to care for chronically ill patients resulting in the better overall assessment of patient’s condition and more intense care for chronically ill
Time savings	n = 32 (13%)	Time savings; concentration on core responsibilities; more time for patients
No relief	n = 11 (5%)	VERAH is not yet employed or used in new capacity; little/no relief; no change because VERAH was already well trained
Category	Entries (n = 189)	Examples of disadvantages
No/very few problems	n = 62 (33%)	Explicitly stated that no problems were associated with employment of VERAH in family practice
Implementation	n = 39 (21%)	Changes to practice structure; team and patients adjust to new situation; employee is no longer available for other tasks; legal uncertainties → questions surrounding legal accountability, e.g. when performing home visits or driving the practice car
Time factor	n = 32 (17%)	Time required for training, when restructuring practice there is no time to adjust to employment of VERAH; VERAH’s hours of work
Costs	n = 25 (13%)	Insufficient compensation and recognition, e.g. by health insurance companies; low salary; lack of staff
Acceptance of VERAHs	n = 23 (12%)	Patients have problems adjusting to new situation; relatives do not accept new situation; patients and colleagues have problems adjusting; doctors have problems adjusting to VERAHs new responsibilities and in delegating tasks
VERAH’s training	n = 8 (4%)	Training content/training requirements/training overly comprehensive; qualification and training of no practical value, excessive administrative burden; in appropriate subject matter; not all training content could be put into practice

### Greatest difficulties associated with employment of VERAHs

62 of 189 responses specified that the employment of VERAHs in the family practice did not cause any problems. 39 described various problems that arose as a result of employing VERAHs in the practice (changes to the structure of the practice, difficulties experienced by patients in adapting to the new situation and legal uncertainty). The amount of time that VERAHs required to train for and adjust to their new function was also mentioned as a difficulty in 32 cases. Further problems resulted from the reluctance of health insurance companies to provide sufficient compensation and respect for the work performed by VERAHs (n = 25), a lack of acceptance of VERAHs by patients, their relatives and non-physician colleagues (n = 23), and the excessive demands made on VERAHs in order to obtain the new qualification (n = 8) (see Table 3).

## Discussion

Whereas most of the more simple tasks, such as vaccinations, wound management or measurement of diagnostic parameters, were assigned to all HCAs irrespective of their training, some tasks stand out as tasks that are mainly delegated to the specially trained staff (VERAHs). The tasks that were delegated to VERAHs by the majority of GPs were home visits (59%), the assessment of physical health as part of a comprehensive assessment of a patient’s condition (63%), and the preparation of a care plan (58%). About half the GPs (49%) indicated that they were willing to delegate case management assessments to their VERAHs (31% delegate case management assessments to all their staff and 20% do not delegate them at all (see Table 2).

Only one medical task is regarded by half the GPs as being the sole responsibility of a GP: 46% said the assessment of mental health as part of the comprehensive assessment of a patient’s condition was something they preferred to do themselves. One reason for this may be that it is difficult for non-mental health specialists to recognize [15] and it is therefore particularly difficult for GPs to entrust such tasks to their MFAs and VERAHs.

Our study shows that in a country in which shifting responsibility to non-physician practice staff has only recently been introduced, and non-physician staff are expected to have no more than 3 years of training, delegation as a concept has nevertheless been accepted. The results show that VERAHs are mainly employed in rural practices where the demand for health care personnel is expected to be greatest in the future. However, some physicians remain reluctant to assign these tasks either to HCAs or to VERAHs (see Table 2). Our results may reflect a system in which team-based care is only slowly being integrated into the legal framework.

On the other hand, our study showed that even tasks entailing substantial responsibility were delegated to the specially trained VERAHs. In particular, responsibility for home visits and the structured assessment of a patient’s physical well-being, as well as the assessment and preparation of care plans as part of case management, are generally delegated to VERAHs. It is worth mentioning that VERAHs are specifically trained to carry out home visits and case management. It would appear that in a “lone-doctor-with-helpers”-world a prerequisite for the delegation of tasks is the targeted training of HCAs that have been working in the practice for several years. Interestingly, the HCAs relieved the perceived burden on GPs by taking responsibility for many different kinds of task, including the assessment of a patient’s condition. However, the employment of a VERAH in the family practice also means providing her with the time to pursue her training and accepting other time constraints resulting from changes in the practice. Special training to become a VERAH provides practices with a cost-effective means of qualifying their personnel but it does require that practices are prepared to redistribute certain tasks. Changes to compensation models should also reflect the new distribution of responsibilities within the general practice team.

The VERAH qualification has been specifically designed to meet the needs of general practices. So far, around 8,000 persons have qualified to become VERAHs in Germany. In other countries, specially qualified non-physician medical personnel are more common, such as primary healthcare nurse practitioners in Canada [16], or nurse practitioners and practice nurses in the U.S. [17], or in Australia [18]. These highly-trained primary care health professionals generally have a bachelor’s or master’s degree.

International comparisons show that in many countries (United States, Canada, Australia, England and the Netherlands) primary care has long been delivered by teams of physicians and other health care professionals [19]. In these countries it appears to be considered perfectly normal that complex tasks are carried out by different categories of medical professional, depending on their qualifications. In Germany, the general practitioner has been solely responsible for providing medical care in his or her practice up to now. Nonetheless, we have seen that general practitioners are willing to shift responsibility for certain relatively difficult and complex tasks to specially qualified personnel.

The possibility to delegate complex tasks such as home visits is a major reason to employ a VERAH, as it leads to considerable time savings for the doctor. Home visits are a central element of primary care for patient groups that are immobile and bound to their living environments [20–21]. Up to several years ago, the situation was generally regarded as unsatisfactory due to increasing time constraints, but German GPs could not envisage any alternative that would enable them to reduce their workload [22].

Whereas in many countries, specially trained non-physician personnel have long been involved in decision making, the profession of HCA in Germany generally entails taking responsibility for administrative work and simple tasks in practice-based health care (e.g. performing an ECG, answering the telephone etc.) [23]. Even in 2006, of the 62% of family doctors that employed an HCA, only 15% said they could imagine extending their responsibilities and allowing them to play a more active role in the care of patients [24].

Our results reflect a trend that has been taking place in the U.S. for some time now [25]. Medical assistants in the U.S. have become increasingly involved in the care of patients. They observe the course of treatment, remind patients of necessary examinations, ask about their patients’ needs and document important findings before the patient sees the doctor. In this way, medical assistants work as patient coaches and can, for example, influence the behavior of diabetics [26]. Considering the shortage of doctors in the U.S., patients have been happy to accept the greater role now played by medical assistants in their care [27].

On the other hand, Halter et al. [28] point out in a systematic review of international studies, that it is not always possible to compare the situation in different health care systems. Physician assistants in the U.S., for example, are qualified to perform many more tasks (e.g. they can prescribe medications) than in Germany. The question what is and can be delegated often depends on who is legally accountable for the outcomes of health care: it is difficult to delegate tasks to non-physician staff for which they are not ultimately accountable. Nevertheless, Halter et al. agree that in view of the growing number of non-physician health care professions and an expected shortage of doctors in the future, the greater use of this resource requires further study [27]. For the German health care system it is also necessary to investigate whether the involvement of VERAHs actually helps reduce the workload on doctors and how the freed-up resources are used.

### Strengths and limitations

This study is based on a survey that was conducted as part of the evaluation of a structured health plan that trains HCAs to become VERAHs. The plan also foresaw an increase in budgets to reflect that VERAHs are qualified to take responsibility for the care of chronically ill patients. Every practice was free to decide which tasks should continue to be performed by the GPs, which should be conducted by VERAHs and which by HCAs.

Hence, the results reflect a willingness to shift responsibility within the practice team, independently of any formal requirements regarding the tasks that should be delegated. On the other hand one limitation of the study is that only GPs were surveyed, and only those that already employed a VERAH. It is thus possible to gauge the experiences of GPs that employ VERAHs but not possible to draw conclusions as to the tasks GPs generally consider suitable for delegation. From a former study we know that GPs that employ VERAHs are generally about 5 years older, male (84% vs. 47%) and have 6 more years of working experience than GPs who employ HCAs without an additional qualification. Initially, we invited all GPs that were participating in the structured health plan and employing a VERAH to take part in the study, and received answers from about 26% of them. The results may therefore reflect task delegation in practices where delegation has proved worthwhile, as GPs might otherwise not have felt inclined to answer this questionnaire.

The survey took place in a system of primary health care in which the family physician is solely accountable for what happens in his or her practice and HCAs have usually received only limited training. The results of our survey show what tasks are generally delegated and to whom (as well as which are not delegated at all), and describe the reasons for this, along with the advantages and disadvantages associated with shifting responsibilities in such a health care system.

## Conclusions

In Germany, where GPs are solely accountable for the health care provided in their practices, experience with the transfer of responsibility to other non-physician health care personnel is very limited. Interestingly, only the assessment of mental health as part of the comprehensive assessment of a patient’s condition continues to be carried out by almost half the GPs themselves. A large number of GPs are happy for VERAHs to take on greater responsibility, even for complex tasks, and to play a more active role in the provision of health care. Tasks that involve a substantial degree of know-how and that the surveyed doctors were willing to delegate to VERAHs include home visits and case management. This “new” role allocation within the practice may signal a shift in the provision of health care to more team-oriented care in Germany. However, HCAs that have received no special training continue for the most part to carry out the tasks for which they have traditionally been trained.

## Supporting Information

S1 File Questionnaire for the GPs. (PDF)
