# Supplementary material for: Sharing Responsibilities within the General Practice Team – A Cross-Sectional Study of Task Delegation in Germany
Source: PLoS One. 2016 Jun 9;11(6):e0157248. doi: 10.1371/journal.pone.0157248 (PMC4900540; doi:10.1371/journal.pone.0157248)
Supplement: S1 File — (PDF) [file pone.0157248.s001.pdf]

Questionnaire for GPs participating in the study

"Evaluation of the employment of VERAHs as part of a structured health plan in Baden-Wuerttemberg"

Please mark your answer by placing a cross in the relevant box. You may cross more than one box if more than one answer is applicable.

1. Why did you decide to employ a VERAH in your practice?

- ☐ Possibility to delegate home visits
- ☐ Possibility to improve the health care of chronically ill patients
- ☐ Possibility to save time
- ☐ Other: ____

The following questions concern changes in the practice that you have noticed since employing a VERAH. Please respond to each of the statements (please mark only one box in each line).

**2. Since a VERAH has worked in the practice …**	Agree	Neither agree nor disagree	Disagree
… when making decisions related to patient health care I have involved her more than before she qualified as a VERAH	☐	☐	☐
… delegating work to her has enabled me to save a significant amount of time	☐	☐	☐
… I can make better use of practice team resources now	☐	☐	☐
… our chronically ill patients have benefited most from the VERAH's work	☐	☐	☐

The following questions concern the delegation of specific tasks to VERAHs. Which of the following do you delegate exclusively to VERAHs, which to other HCAs/practice personnel and which do you carry out yourself? (Please mark only one box in each line)

**3. Tasks delegated …**	…mainly to VERAHs	…to all staff	Not delegated at all
Vaccination management (e.g. preparing vaccination plans, explaining reasons for vaccinations etc.)	☐	☐	☐
Medication management (e.g. comparing prescribed medications with medication packaging)	☐	☐	☐
Patient training (e.g. diabetes management, nutrition advice etc.)	☐	☐	☐
Wound management (e.g. changing bandages in case of leg ulcers etc.)	☐	☐	☐

**3. Tasks delegated …**	…mainly to VERAHs	…to all staff	Not delegated at all
Carrying out diagnostic tests<br>(e.g. blood glucose etc.)	☐	☐	☐
Structured assessments of a patient's physical condition<br>(e.g. when VERAH visits a patient at home)	☐	☐	☐
Structured assessment of a patient's mental condition	☐	☐	☐
Symptoms of complaint and/or disease<br>(e.g. using a structured questionnaire etc.)	☐	☐	☐
Documentation of medically significant facts (e.g. falls, medication-related problems, changes in social environment etc.)	☐	☐	☐
Simple medical procedures<br>(e.g. taking blood samples, injections)	☐	☐	☐
Assessments (holistic assessment of a patient's health status and living conditions including problems and resources, for example on basis of questionnaire)	☐	☐	☐
Re-assessments (monitoring)	☐	☐	☐
Preparation of care plan	☐	☐	☐
Dialog with other institutions<br>(e.g. welfare services, nursing services)	☐	☐	☐
Patient support in coordination and organization of treatment (e.g. by consulting other doctors or going to other health care institutions)	☐	☐	☐
Home visits	☐	☐	☐
Geriatric assessments<br>(e.g. assessment of danger of falls, mini mental status exam, dementia test etc.)	☐	☐	☐

4. What are the main advantages of employing a VERAH?

___

___

___

5. What are the main disadvantages of employing a VERAH?

___

___

___

6. Sex

☐ female ☐ male

7. Year of birth: ☐☐☐☐

8. Years in private practice

Since ☐☐ years

9. Previous participation in research project?

☐ Yes ☐ No

10. Family practices:

☐ Solo practice

☐ Joint practice

11. Practice personnel

Number of doctors in practice ☐☐

Number of non-physician staff ☐☐

12. Location of practice

☐ City with more than 100,000 inhabitants

☐ Town with 20,000 to 100,000 inhabitants

☐ Rural town with 5,000 to 20,000 inhabitants

☐ Village with less than 5,000 inhabitants

Thank you very much for participating!
